# Enhanced spectral response in frequency‐dependent diffusion measurements using a linear encoding model

**DOI:** 10.1002/mrm.70006

**Published:** 2025-07-28

**Authors:** Eric Seth Michael, Franciszek Hennel, Klaas Paul Pruessmann

**Affiliations:** ^1^ Institute for Biomedical Engineering, ETH Zurich and University of Zurich Zurich Switzerland

**Keywords:** diffusion MRI, frequency‐dependent diffusion, oscillating gradient, spectral response

## Abstract

**Purpose:**

To devise a more comprehensive quantitative representation for spectral encodings in frequency‐dependent diffusion measurements for improved estimation of *D*(ω).

**Theory and Methods:**

Whereas a spectral diffusion measurement is typically represented by a Dirac delta function at a single attributed frequency, spectral response is represented here by the encoding power in |*Q*(ω)|^2^ over a set of contiguous frequency intervals. Using this representation paradigm, a linear encoding model is formulated wherein diffusivity over each interval can be estimated by inverting the encoding process from a set of measurements. This strategy was validated in in vivo human brain imaging experiments evaluating *D*(ω) up to 50 Hz over 10‐Hz intervals using high‐performance gradients. The employed spectral encodings were selected using an accompanying framework devised to ensure robust encoding performance given the chosen frequency intervals. Additionally, simulated measurements were carried out to compare the performance in estimating *D*(ω) using the proposed encoding model versus using single‐frequency attribution in relation to the form of *D*(ω) and the width of frequency intervals.

**Results:**

In vivo *D*(ω) determined using the proposed encoding strategy were found to increase with increasing frequency and could be mapped to spectral responses more spectrally selective than those characteristic of single‐frequency attribution. In turn, simulated measurements demonstrated that the linear encoding model permitted *D*(ω) estimation with improved accuracy, especially for more nonlinear *D*(ω), at the expense of reduced precision, particularly for narrower frequency intervals.

**Conclusion:**

By devising a more holistic representation paradigm for frequency‐dependent diffusion measurements, *D*(ω) can be recovered with higher fidelity.

## 1 INTRODUCTION

Diffusion MRI permits noninvasive characterization of tissue microstructure by encoding the Brownian motion of water molecules, which is shaped by the microenvironment. The temporal frequency spectrum of diffusion, *D*(ω), introduces a valuable dimension to this measurement space that reflects microstructural characteristics over different length scales. Evaluation of this frequency dependence, a technique known as *temporal diffusion spectroscopy* (TDS),^1^ provides insight into microstructural disorder^2^ and can be used to determine clinically relevant microstructural properties, such as cellular density in tumors^3^ and axon diameter.^4^

Most TDS approaches employ a series of different diffusion encodings, each tailored to probe and attributed to a single, unique frequency. In such experiments, spectrally selective diffusion encoding is achieved using monopolar pulsed gradient (PG) and cosine‐modulated trapezoidal oscillating gradient (OG) waveforms. PGs yield encoding spectra peaked at 0 Hz and are accordingly deemed to sample 0 Hz. OGs yield largely unimodal encoding spectra peaked near the gradient oscillation frequency and are considered to sample the peak or centroid frequency of the encoding spectrum or the nominal oscillation frequency of the gradient waveform. Spectral encodings with less conventional lineshapes—namely, exhibiting multimodal selectivity, as in sine‐modulated OG waveforms^5^—can also be employed but have not gained much traction due the more straightforward interpretation of spectrally pure lineshapes. An alternative approach to TDS based on Fourier encoding uses harmonically modulated encoding spectra but struggles to attain ample diffusion encoding strength.^6^

**FIGURE 1:**
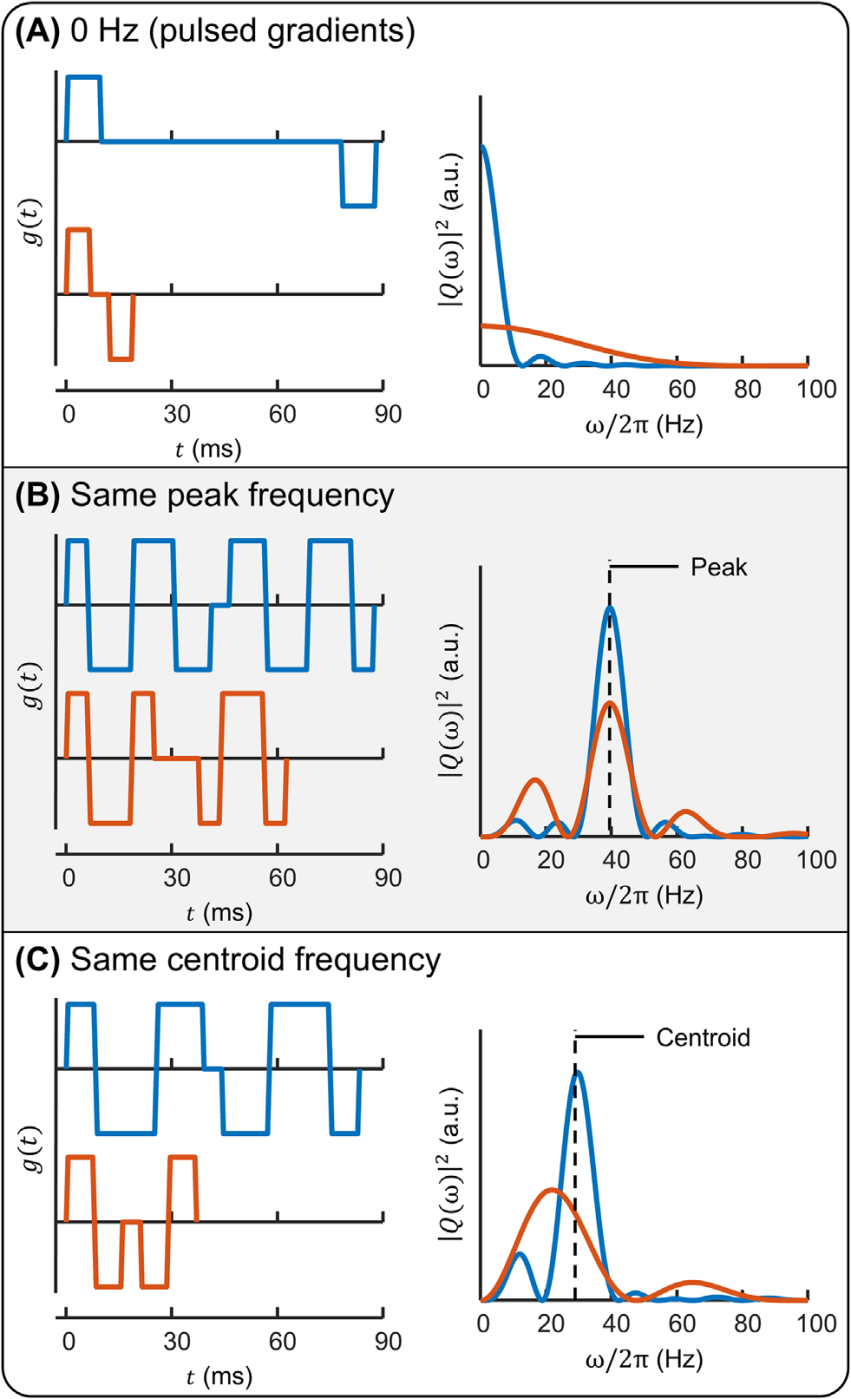
Ambiguity in representing the spectral response of a diffusion gradient waveform based on the 0‐Hz convention for PGs (A), the peak frequency (B), and the centroid frequency (C). For each case, two gradient waveforms producing the same frequency assignment are shown. All *g*(*t*) are shown with the same amplitude in order to highlight relative temporal characteristics. Encoding spectra, |*Q*(ω)|^2^, within each subfigure are scaled to have the same integral to draw a comparison in which the same b‐value is achieved. PG, pulsed gradient.

The common practice of representing each diffusion encoding by a single frequency (i.e., as a Dirac delta encoding) renders the fidelity of frequency‐dependent diffusion measurements limited by the correspondence of the spectral responses to the attributed frequencies. Consistency in this regard is best achieved by improving the design of gradient waveforms to enhance spectral characteristics.^7^, ^8^, ^9^, ^10^ However, single‐frequency attribution still often amounts to an oversimplification of spectral lineshapes. For instance, PG spectral bandwidth and sidelobe behavior can widely vary depending on pulse width and spacing but are neglected by attributing all cases—representing different effective diffusion times—to 0 Hz (Figure 1A),^11^ and distinct OG encoding spectra (e.g., in terms of bandwidth, symmetry, or sidelobe behavior) can have identical peak or centroid frequencies (Figure 1B,C). As a result of these ambiguities, encodings assigned to the same frequency may yield different diffusivity measurements, rendering spectrally derived microstructural metrics vulnerable to systematic inaccuracies.

In light of these limitations in TDS, the goal of this work was to devise a more holistic representation of encoding spectra to facilitate better quantification of *D*(ω). To this end, we propose to represent spectral response in TDS measurements by spectral power over a set of contiguous frequency intervals chosen by the user. In turn, diffusivity can be estimated per frequency interval provided that the series of spectral diffusion measurements has adequately encoded the intervals. To fulfill this condition, we also present a framework for selecting a set of diffusion gradient waveforms / encoding spectra that robustly encodes the chosen set of frequency intervals on the basis of noise propagation and spectral contamination in the ultimate interval‐wise diffusivity estimates.

The proposed methodology is validated by estimating *D*(ω) over 10‐Hz intervals up to 50 Hz in the in vivo human brain, for which *D*(ω) obtained using single‐frequency attribution served as a reference. These results are juxtaposed with a series of simulated measurements of several physically plausible diffusion spectra to compare *D*(ω) estimation using the proposed versus conventional representation paradigm in relation to the form of *D*(ω) and the width of the chosen frequency intervals.

## 2 THEORY

Under the Gaussian phase approximation,^12^ the signal attenuation resulting from diffusion encoding can be expressed in the temporal frequency domain as

(1) $$\ln\left(\frac{S_{0}}{S}\right)=\frac{1}{\pi }\int_{0}^{\infty }D\left(\omega \right){\left|Q\left(\omega \right)\right|}^{2}d\omega ,$$

where *S* and *S*_0_ are signals with and without diffusion encoding, respectively, *D*(ω) is the frequency‐dependent diffusivity, and |*Q*(ω)|^2^ is the diffusion encoding power spectrum. *Q*(ω) is the Fourier transform of the q‐space trajectory, *q*(*t*),

(2) $$Q\left(\omega \right)=\int_{-\infty }^{\infty }q\left(t\right)e^{i\omega t}\mathrm{dt},$$

where *q*(*t*) is the time integral of the diffusion‐sensitizing gradient waveform, *g*(*t*),

(3) $$q\left(t\right)=\gamma \int_{0}^{t}g\left(t^{\prime }\right)dt^{\prime }.$$

**FIGURE 2:**
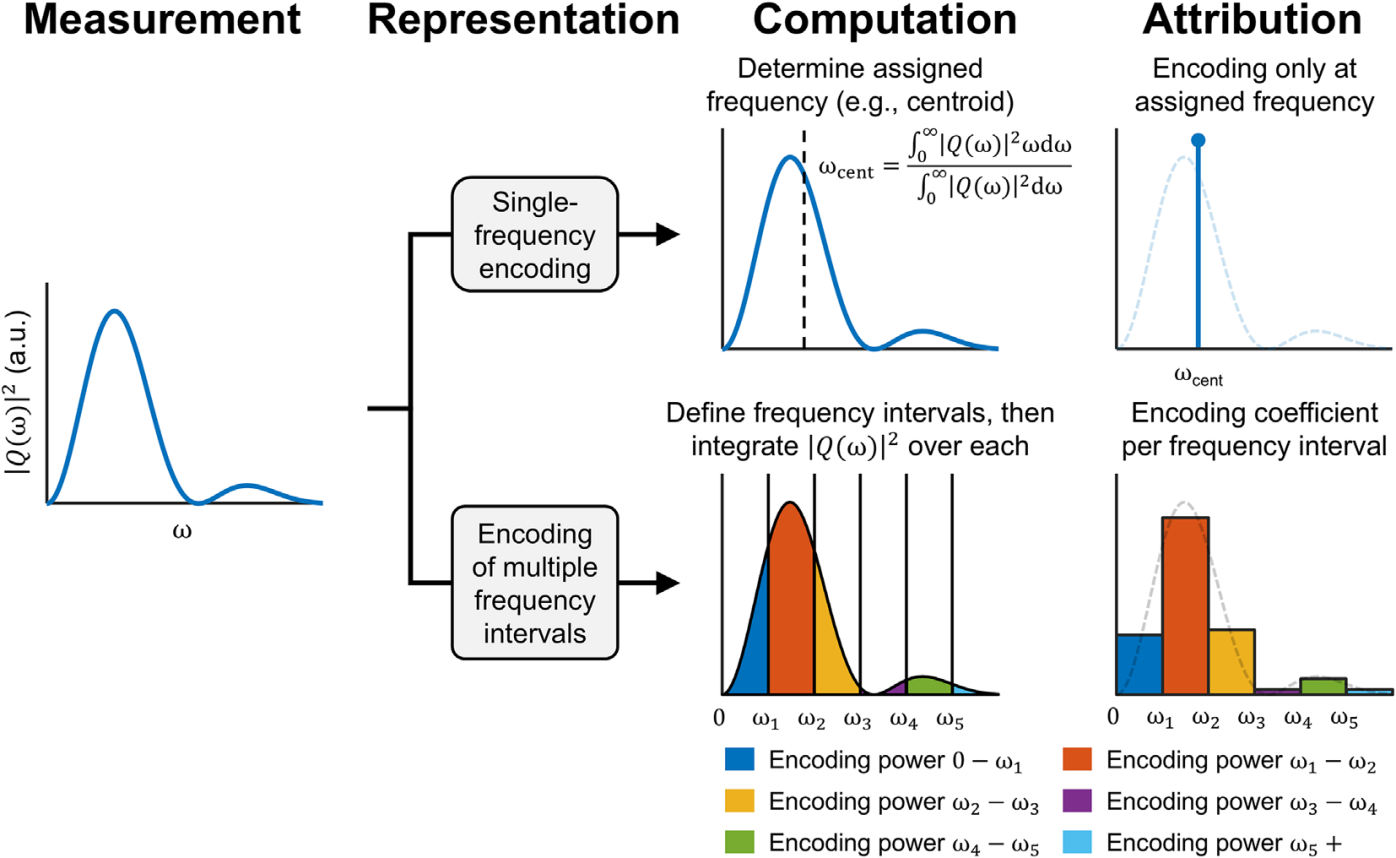
Comparison of measurement representations in temporal diffusion spectroscopy. The first column illustrates an example encoding spectrum, |*Q*(ω)|^2^, which characterizes a spectral diffusion measurement. The top row in subsequent columns represents conventional single‐frequency attribution in which the measurement is represented by a Dirac delta encoding at the assigned frequency. The bottom row presents an alternative representation in which the encoding spectrum is more holistically accounted for. In this alternative, a series of frequency intervals must first be defined, after which the encoding spectrum is integrated over each interval; this integration produces an encoding coefficient per interval. Subsequently, the measurement can be represented by a simultaneous encoding of all frequency intervals weighted by the respective encoding coefficients.

By subdividing the frequency axis in Eq. 1 into *N* contiguous intervals, the attenuation can be rewritten as

(4) $$\begin{array}{cc} \ln\left(\frac{S_{0}}{S}\right) & =\frac{1}{\pi }(D_{1}\int_{0}^{\omega_{1}}{\left|Q\left(\omega \right)\right|}^{2}d\omega +D_{2}\int_{\omega_{1}}^{\omega_{2}}{\left|Q\left(\omega \right)\right|}^{2}d\omega \\ & \;+\ldots +D_{N}\int_{\omega_{N-1}}^{\infty }{\left|Q\left(\omega \right)\right|}^{2}d\omega ), \end{array}$$

where *D*_*n*_ and $\frac{1}{\pi }\int_{\omega_{n-1}}^{\omega_{n}}{\left|Q\left(\omega \right)\right|}^{2}d\omega$ represent diffusivity and encoding power, respectively, over the *n*th frequency interval from ω_*n*−1_ to ω_*n*_, and *n* enumerates intervals; the final frequency interval has infinite width, which will be addressed later. According to this reformulation, a spectral diffusion measurement can be viewed as a simultaneous encoding of all frequency intervals, where each interval is encoded in proportion to its spectral encoding power. This measurement representation is visualized and compared to conventional single‐frequency attribution in Figure 2.

As a consequence of the proposed representation, diffusivity cannot be uniquely determined over any frequency interval by performing a single measurement; this principle is contrary to a spectral measurement attributed to a single frequency, for which diffusivity at the assigned frequency is determined by the measurement. Determination of all diffusivities, then, requires a series of *M* ≥ *N* spectral diffusion measurements, each with a different |*Q*(ω)|^2^, which acts as the principal experimental variable. Subsequently, the series of spectral diffusion measurements can be posed as a linear system of equations, **S** = **ED**: **S** is the *M* × 1 signal vector,

(5) $$S={\left[\begin{array}{lll} \ln\left(\frac{S_{0}}{S_{1}}\right) & \cdots & \ln\left(\frac{S_{0}}{S_{M}}\right) \end{array}\right]}^{T},$$

where *S*_1_ through *S*_*M*_ are the *M* diffusion‐weighted signals, and T denotes the transpose; **E** is the *M* × *N* encoding matrix, for which

(6) $$E_{m,n}=\frac{1}{\pi }\int_{\omega_{n-1}}^{\omega_{n}}{\left|Q_{m}\left(\omega \right)\right|}^{2}d\omega ,$$

where *m* enumerates measurements, and |*Q*_*m*_(ω)|^2^ is the encoding spectrum used in the measurement of signal *S*_*m*_; and **D** is the *N* × 1 diffusivity vector,

(7) $$D={\left[\begin{array}{lll} D_{1} & \cdots & D_{N} \end{array}\right]}^{T}.$$

The unknown diffusivities can then be determined using the pseudoinverse of the encoding matrix, which is given by

(8) $$R={\left(E^{T}E\right)}^{-1}E^{T}$$

and has dimensions *N* × *M*; this matrix, **R**, is also known as the reconstruction matrix. Using **R**, the diffusivity estimates can be computed as

(9) $$\hat{D}=\mathrm{RS}.$$

We propose to implement this linear encoding method in two steps: (1) define the frequency intervals over which diffusivity should be estimated; then (2) select the set of |*Q*(ω)|^2^s to apply. Crucially, the |*Q*(ω)|^2^s should be chosen such that all frequency intervals are sufficiently encoded, thereby permitting reliable estimation of each diffusivity, and should all have the same total spectral power (i.e., b‐value) in order to balance potential sources of bias (e.g., kurtosis, perfusion) across measurements.

## 3 METHODS

### 3.1 Selection of frequency intervals

The selection of frequency intervals to employ in the linear encoding model has three primary parametric constraints: (1) the maximum available gradient amplitude, *G*_max_; (2) the desired b‐value, *b*; (3) the maximum allowable diffusion‐encoding duration, *T*_DW_. Together, these three parameters dictate the maximum practical gradient oscillation frequency and, consequently, the feasible encoding range according to $\omega_{\max}\propto G_{\max}T_{\mathrm{DW}}^{1/2}b^{-1/2}$.^5^ Furthermore, the minimum achievable spectral bandwidth of a diffusion‐sensitizing gradient waveform is limited by *T*_DW_ as given by FWHM ≈ 0.9/*T*_DW_.^7^ Based on available gradient hardware and the target application of in vivo human brain imaging, the following specifications were made: *G*_max_ = 200 mT/m,^13^ *b* = 1000 s/mm^2^, and *T*_DW_ ≤ 90 ms. Consequently, primary frequency intervals with widths of 10 Hz up to 50 Hz were chosen for in vivo imaging experiments. To account for encoding power from higher frequencies, an additional interval for frequencies beyond this upper limit (i.e., 50 Hz to ∞) was included. Aggregating all high‐frequency contributions into one interval accounts for the residual encoding power and resulting signal attenuation, thereby precluding aliasing, but does not necessitate additional spectral resolution.

### 3.2 Criteria for selection of encoding spectra

Given the ultimate objective of precise and accurate estimation of diffusivity per interval, two evaluation criteria were used in the selection of encoding spectra: noise propagation, and the spectral response of the frequency intervals.

Given a set of encoding spectra, the propagation of noise can be quantified using the corresponding reconstruction matrix defined in Eq. 8. Assuming the same noise level and uncorrelated noise across measurements (i.e., all ln(*S*_0_/*S*_*m*_) in **S**), noise amplification with respect to the measurement noise level, σ^S^, can be expressed as

(10) $$\sigma_{n}^{D}=\sigma^{S}\sqrt{{\left(RR^{T}\right)}_{n,n}},$$

where $\sigma_{n}^{D}$ is the noise level of the diffusivity estimate of the *n*th frequency interval.

Regarding spectral response, the linear encoding model implicitly represents frequency intervals by boxcar functions. An interval's actual spectral characterization, however, is governed by its spectral response function (SRF), which is the spectral distribution of diffusivities contributing to the frequency interval. This quantity is analogous to the *spatial* response function used in imaging.^14^ SRFs can be computed as

(11) $${\mathrm{SRF}}_{n}\left(\omega \right)=\sum_{m}R_{n,m}{\left|Q_{m}\left(\omega \right)\right|}^{2},$$

where SRF_*n*_(ω) denotes the SRF of the *n*th frequency interval. As such, the effective response function of each frequency interval is a linear combination of the prescribed encoding spectra, parallel to the linear combination of measurements used to determine each diffusivity as in Eq. 9, and only has a non‐zero interval‐wise sum over the represented interval. It is worth noting that the response functions in single‐frequency attribution are simply the employed |*Q*(ω)|^2^s. To avoid ambiguity, the acronym “SRF” will only be used hereafter in reference to response functions resulting from the linear encoding model.

Joint evaluation of both criteria was facilitated by defining an objective function with which sets of |*Q*(ω)|^2^s could be assessed. Considering that noise propagation should be minimized and SRFs should have minimal contributions outside of the targeted frequency interval, a suitable objective function for minimization is

(12) $$\begin{array}{cc} O\left({\left|Q_{1-M}\left(\omega \right)\right|}^{2}\right) & =\left(\sum_{n=1}^{N}\sqrt{{\left(RR^{T}\right)}_{n,n}}\right)\\ & \;\times \left(\sum_{n=1}^{N}\int_{0}^{\omega_{n-1}}{\mathrm{SRF}}_{n}^{2}\left(\omega \right)d\omega +\int_{\omega_{n}}^{\infty }{\mathrm{SRF}}_{n}^{2}\left(\omega \right)d\omega \right), \end{array}$$

where ${\left|Q_{1-M}\left(\omega \right)\right|}^{2}$ denotes the set of *M* encoding spectra under evaluation. In effect, this function evaluates noise amplification and spectral contamination, given by SRF power outside of the intended frequency interval, for all intervals.

### 3.3 Framework for selection of encoding spectra

Encoding spectra for in vivo imaging were selected using a combinatorial optimization framework for which the objective function in Eq. 12 was the basis for evaluation. This approach was employed to circumvent manually fine‐tuning diffusion gradient waveforms for optimized encoding performance, a process that would be both laborious and unlikely to yield an optimal selection given the multifaceted nature of the objective function.

First, a library of |*Q*(ω)|^2^s corresponding to experimentally feasible diffusion gradient waveforms was created. To generate this library, gradient waveforms parameters (e.g., oscillation frequency for OG, Δ for PG) were iteratively incremented within the experimental specifications stated in Section 3.1, with the resulting waveforms / encoding spectra added to the library. This process amounted to 1208 unique realizations of waveforms / encoding spectra. In addition to conventional PG and gap‐filled OG waveforms,^8^ the library included double‐bipolar gradient waveforms, which can be thought of as 0.5+0.5‐period OG waveforms and can yield centroid frequencies less than 10 Hz. Additionally, waveforms with both matched and unmatched pulse polarities^7^ were generated when applicable to increase the variety of |*Q*(ω)|^2^s in the library. Further details about the library are provided in Supporting Information.

Subsequently, subsets of this library yielding the minimum value of the objective function were determined for different numbers of measurements, *M*, by iteratively whittling down the library of |*Q*(ω)|^2^s using a greedy algorithm as follows:

1. Each iteration begins with *M* encoding spectra (i.e., *M* = 1208 in the first iteration).
2. The objective function is evaluated for all subsets of *M* − 1 encoding spectra.
3. The subset achieving the minimum objective value is kept, representing the locally optimal set of *M* − 1 encoding spectra. The next iteration begins with this optimal set.

**FIGURE 3:**
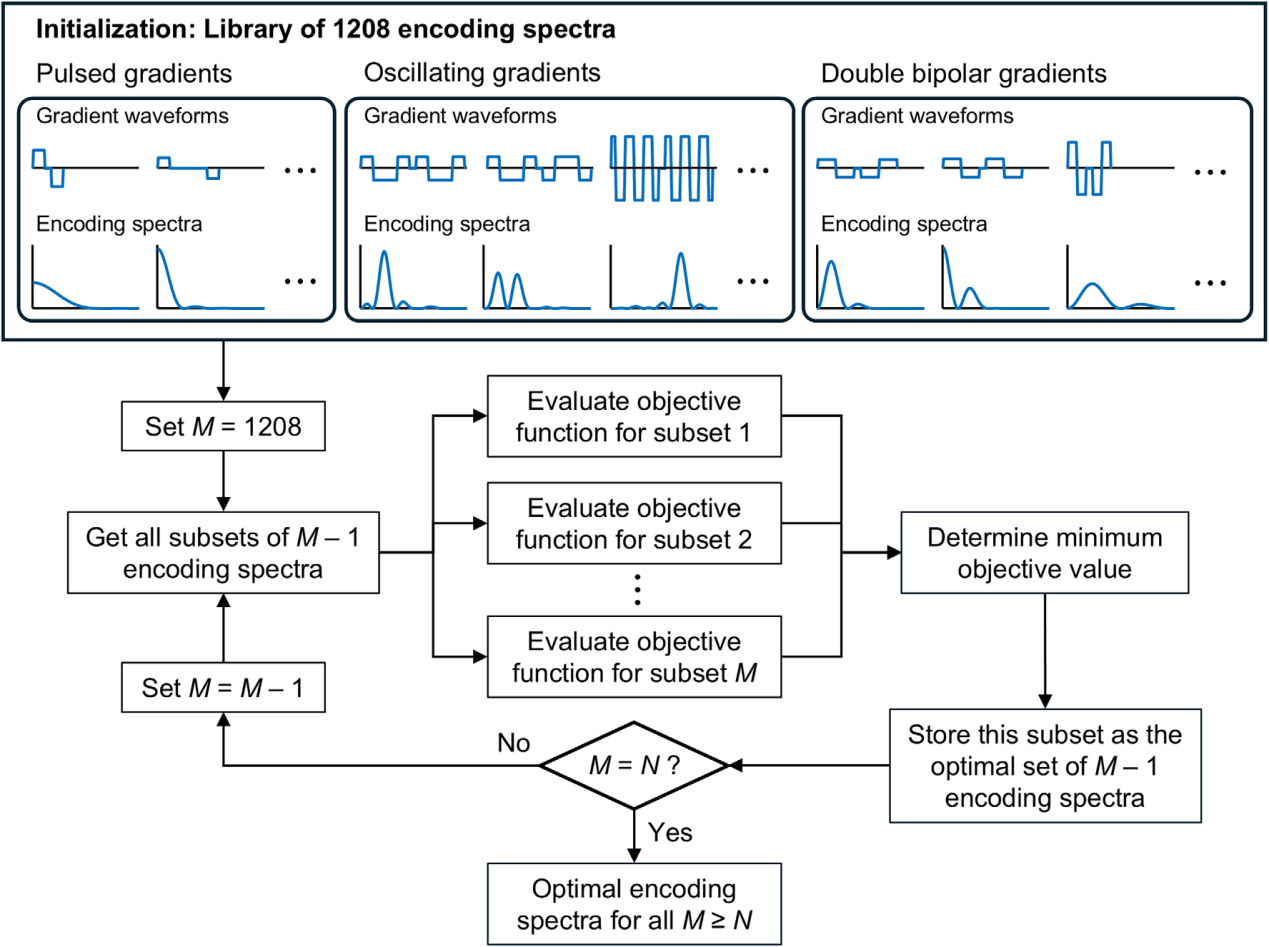
Greedy algorithm for determining optimal encoding spectra to use in frequency‐dependent diffusion measurements. The algorithm is predicated on the linear encoding model for temporal diffusion spectroscopy described in this work and presupposes a set of *N* frequency intervals defined in advance. Under this condition, the algorithm is initialized by a library of experimentally feasible encoding spectra; the library accumulates various realizations of three types of diffusion gradient waveforms. Next, all subsets of this library in which one element is excluded are formed, and the objective function (Eq. 12) is computed for each subset. The subset producing the lowest objective value is considered to be the optimal subset for the number of encoding spectra contained within. The process is then repeated, beginning with the optimal subset just determined: again, all subsets with one element excluded are evaluated. This procedure repeats until the number of remaining encoding spectra is equal to the number of frequency intervals. The result of the algorithm is an optimal set of encoding spectra (among the contents of the library) for different possible numbers of measurements, *M*.

This algorithm is illustrated in Figure 3. A variant of this algorithm was also performed in which the first iteration selected the two (out of 1208) encoding spectra producing the minimum objective value, and subsequent iterations expanded this selection by evaluating all sets containing one of the remaining |*Q*(ω)|^2^s. The subset corresponding to the lowest objective value among both forms of the algorithm was kept for each *M*.

### 3.4 Selection of encoding spectra

For the final selection of encoding spectra for experimentation, the algorithm output for *M* = 6 was considered as the default option, representing the minimum required number of measurements. Performing additional measurements (i.e., using the optimal encoding spectra for *M* > 6) would reduce the overall diffusivity estimation uncertainty and spectral contamination but should be weighed against the resulting increase in measurement time, which is not considered by the objective function. Ultimately, the optimal encoding spectra for *M* = 7 were selected; in particular, the marked improvement in the SRF of the 10–20 Hz interval going from *M* = 6 to *M* = 7 was judged to warrant the additional measurement time. Further increments in *M* yielded less substantial SRF improvements and only minor reductions in noise amplification. A comparison of these evaluation criteria for *M* = 6 through 9 is presented in Figure S1.

### 3.5 Data acquisition

Six healthy adults volunteered for scanning in accordance with applicable ethics policy. Scanning was performed using a Philips 3T Achieva system (Philips Healthcare, Best, the Netherlands) equipped with a high‐performance gradient insert coil^13^ reaching *G*_max_ = 200 mT/m and SR_max_ = 600 mT/m/ms and a transmit–receive quadrature birdcage coil.^15^

**FIGURE 4:**
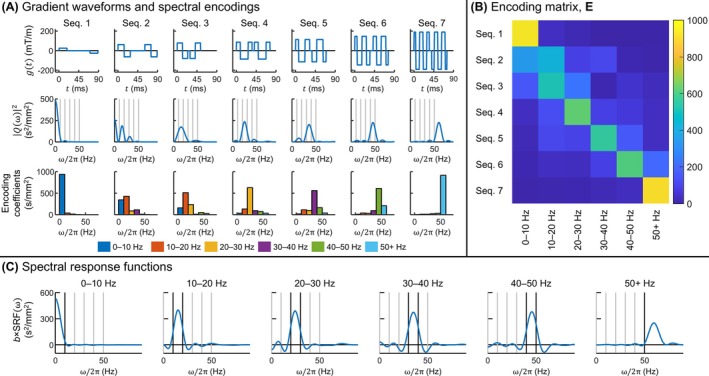
Encoding properties of diffusion gradients used in experiments. (A) Gradient waveforms, encoding spectra, and encoding coefficients for the linear encoding model as per Eq. 6. The vertical lines in the plots of encoding spectra identify edges of the frequency intervals for which the encoding coefficients are shown beneath. Encoding coefficients are illustrated as bars over the entire frequency intervals they represent, except for the final interval, for which the bars have the same widths as other intervals for ease of visualization. Gradient waveforms include the impact of the refocusing RF pulse. (B) Encoding matrix corresponding to the diffusion encodings shown in (A). Each entry in the encoding matrix directly corresponds to an encoding coefficient shown in (A). (C) SRFs for the six frequency intervals. Black vertical lines identify the edges of the frequency interval for which each SRF is shown, whereas gray vertical lines identify other frequency interval edges. SRFs are scaled by the b‐value to achieve equivalent signed spectral areas to the encoding spectra in (A). SRF, spectral response function.

For each volunteer, seven spin‐echo DTI sequences were performed, corresponding to the seven optimal encoding spectra / gradient waveforms: one PG, two double‐bipolar gradients, and four OGs. The gradient waveforms and encoding spectra are shown in Figure 4A. All DTI sequences employed spiral readouts and had 16 diffusion directions, 5 *b* = 0 averages, spatial resolution = 2 × 2 × 3 mm^3^, FOV = 220 × 220 × 96 mm^3^, and TR/TE = 94/6000 ms. In addition, T_1_‐weighted acquisitions (MPRAGE) were performed using the same FOV as in DTI and 0.5 × 0.5 × 1 mm^3^ spatial resolution.

A cylindrical water phantom doped with NaCl and CuSO_4_ was also scanned using the DTI sequences for sequence calibration.

### 3.6 Image reconstruction and preprocessing

All DTI acquisitions were repeated with a field camera based on ^1^H NMR field probes^16^ (Skope Magnetic Resonance Technologies, Zurich, Switzerland) placed inside the scanner in order to measure the spatiotemporal field dynamics during readout. Third‐order spherical harmonic field models corrected for second‐order concomitant fields^17^ were fitted to these data and were used in conjunction with off‐resonance maps in an in‐house higher‐order algebraic reconstruction algorithm.^18^ T_1_‐weighted images were directly reconstructed on the scanner.

In‐house reconstructed images were denoised,^19^ corrected for Gibbs ringing,^20^ and normalized to achieve a consistent *b* = 0 signal level,^21^, ^22^ after which the imaging volumes were coregistered per volunteer using 3D rigid‐body transformations. Subsequently, T_1_‐weighted volumes were transformed to coincide with the reconstructed *b* = 0 volumes using symmetric diffeomorphic image registration^23^, ^24^ implemented in ANTs.^25^ Based on these results, segmentation maps of white matter (WM) and gray matter (GM) partial volume estimates were generated using FSL's FAST.^26^ Masks for both tissue types were then computed by thresholding the respective partial volume estimate maps at 95%. Finally, segmentation masks of WM tracts from the ICBM‐DTI‐81 atlas^27^ and regions from the Harvard‐Oxford subcortical structural atlas^28^ were generated by transforming the respective T_2_‐weighted atlas to coincide with the reconstructed *b* = 0 volumes (as described previously), then applying the same transformation to the tract‐ and region‐wise atlas masks.

### 3.7 Data analysis

Diffusion tensors were fitted voxel‐wise to each DTI dataset using the weighted linear least‐squares approach.^29^ These fits accounted for the effects of gradient nonlinearity during diffusion encoding by using maps describing the spatial variation of diffusion gradient direction vectors, which were computed using vendor‐provided gradient field distributions per Cartesian gradient axis.^30^ Additional sequence‐wise differences between the intended and actual b‐value, which have been previously observed for this gradient system,^9^ were also incorporated as determined from the phantom data (see Supporting Information and Figure S2). Subsequently, mean, axial, and radial diffusivity (MD, AD, and RD, respectively) were determined.

The proposed linear encoding model for TDS was then implemented on a voxel‐wise basis to obtain diffusivity maps for the 0–10, 10–20, 20–30, 30–40, 40–50, and 50+ Hz frequency intervals for each volunteer. All seven spectral encodings were used, and entries of the signal vector, **S**, were computed as *b* × *D*_(*i,j*),*m*_, where *D*_(*i,j*),*m*_ is the *i*th row and *j*th column of the diffusion tensor fitted to the *m*th DTI measurement. This formulation is analogous to ln(*S*_0_/*S*_*m*_), which is traditionally equated to *b* × ADC, and allows the linear encoding model to produce diffusion tensors for the given frequency intervals as output. As such, entries of $\hat{D}$ upon executing Eq. 9 could be expressed as *D*_(*i,j*),*n*_, representing diffusion tensor elements for the *n*th frequency interval. After computing diffusion tensors per frequency interval, MD, AD, and RD were computed.

### 3.8 Simulated frequency‐dependent diffusion measurements

To evaluate the estimation performance of the proposed linear encoding model with respect to single‐frequency attribution, simulated spectral diffusion measurements were performed for different assumed *D*(ω) and using encodings optimized for different sets of frequency intervals.

#### 3.8.1 Dependence on *D*(ω)

First, simulated measurements were performed using the same seven spectral encodings as were used in imaging experiments (see Figure 4) and four physically plausible diffusion spectra corresponding to the following theoretical scenarios:

1. Short‐range disorder along one dimension (*D*_0_ = 7 × 10^−4^ mm^2^/s, θ = 0.5, Λ = 8.5 μm^2^/s^0.5^)^2^, ^31^
2. Short‐range disorder in two dimensions (*D*_0_ = 7 × 10^−4^ mm^2^/s, θ = 1, Λ = 0.48 μm^2^)^32^, ^33^
3. Impermeable spherical cells (*r* = 5 μm) in extracellular water (*f*_in_ = 0.5, *f*_ex_ = 0.5, *D*_in_ = 3 × 10^−3^ mm^2^/s, *D*_ex_ = 2.2 × 10^−3^ mm^2^/s)^3^, ^34^
4. Impermeable spherical cells (*r* = 10 μm) in extracellular water (*f*_in_ = 0.5, *f*_ex_ = 0.5, *D*_in_ = 3 × 10^−3^ mm^2^/s, *D*_ex_ = 2.2 × 10^−3^ mm^2^/s)

To evaluate the theoretical accuracy of each measurement representation, the value of ln(*S*_0_/*S*) was computed per combination of |*Q*(ω)|^2^ and *D*(ω) based on Eq. 1; these calculations represent noiseless simulated measurements. Diffusivities per frequency interval were determined using the linear encoding model, and diffusivities at assigned frequencies (0 Hz for PG, centroid frequency for OG and double‐bipolar) were directly given by ln(*S*_0_/*S*)/*b*. Subsequently, accuracies were computed in terms of the percentage error: diffusivities estimated using the linear encoding model were compared to *D*(ω) at the centroid of the respective interval's SRF, whereas diffusivities estimated using single‐frequency attribution were compared to *D*(ω) at the corresponding assigned frequency.

To evaluate precision, 16 diffusion‐weighted and 5 unweighted signals (|*Q*(ω)|^2^ = 0 for the latter) were generated for each combination of |*Q*(ω)|^2^ and *D*(ω) by sampling values from Gaussian distributions, for which the mean *S* was set to 1 and the mean *S*_0_ was scaled in proportion based on Eq. 1. The distributions' SDs were set to the temporal SD of *b* = 0 signal magnitude in WM in a representative volunteer. Diffusivities were estimated for both measurement representations using the average *S*_0_ and *S* per combination in Eqs. 1 and 5; these calculations represent simulated measurements in the presence of noise. The entire procedure from signal generation to diffusivity estimation was repeated 3000 times, after which the SDs of all diffusivity estimates were computed.

Finally, for scenarios 1 and 2, diffusion dispersion parameters *D*_0_ and Λ were estimated from the simulated measurements to evaluate accuracy and precision in model fitting. The two parameters were fitted as the least‐squares solution to *D*(ω) = *D*_0_ + Λω^θ^ over all diffusivity estimates per measurement representation for each repetition of signal generation and diffusivity estimation. Errors were again derived from the noiseless simulated measurements, and SDs were derived from the repeated noisy simulated measurements.

#### 3.8.2 Dependence on frequency interval width

To assess the estimation performance of the proposed encoding framework with respect to the width of frequency intervals, encoding spectra were first selected as described in Sections 3.3 and 3.4 for different interval widths / numbers of intervals, retaining a 50‐Hz maximum for the primary frequency intervals: 2 × 25‐Hz, 3 × 16.7‐Hz, 4 × 12.5‐Hz, and 6 × 8.3‐Hz. All cases also included a 50+ Hz interval. Using the resulting optimal encoding spectra for each *N* (see Figure S3), simulated measurements equivalent to those described in the previous section were performed for the same four diffusion spectra and followed by the same evaluations of accuracy and precision in diffusivity estimates.

## 4 RESULTS

### 4.1 Encoding spectra selection

Figures 4B,C and Table 1 show the encoding matrix, SRFs, and effective noise amplification factors, respectively, corresponding to the TDS linear encoding model using the encoding spectra / gradient waveforms shown in Figure 4A. The encoding matrix indicates that all but the second spectral measurement primarily encode a single, unique frequency interval. This behavior is a byproduct of the noise propagation term in the objective function because amplification of measurement noise in diffusivity estimation reduces as the measurement encoding functions become more orthogonal. Of these six encoding spectra, the average percentage of the encoding power in the primary interval is 70%. The residual interval‐wise spillover results in an average 49% amplification in the noise level of diffusivity estimates with respect to the measurement noise level under the stated assumptions.

**TABLE 1:** Noise amplification coefficient per frequency interval using the diffusion encodings shown in Figure 4A in the proposed linear encoding model.

Interval	$\sigma_{n}^{D}/\left(\sigma^{S}/b\right)$
0–10 Hz	1.07
10–20 Hz	1.88
20–30 Hz	1.76
30–40 Hz	1.97
40–50 Hz	1.79
50+ Hz	1.11

On the other hand, the second spectral measurement primarily encodes two intervals, with 34% and 43% of its encoding power in the first and second frequency intervals, respectively, and is a byproduct of the SRF side lobe term in the objective function. Although unconventional, the bimodal encoding spectrum effectively sharpens the SRF for the 10–20 Hz frequency interval, thereby mitigating the considerable negative departure of this SRF between 0 and 10 Hz with one fewer measurement (Figure S1). Encoding the 10–20 Hz interval is the most difficult in terms of spectral selectivity because unimodal encoding spectra with a peak in this frequency range tend to have an adjacent local minimum at 0 Hz and, therefore, appreciable spillover into the 0–10 Hz frequency interval. Ultimately, the difficulty is resolved by effectively leveraging two measurements to achieve satisfactory spectral response for the 10–20 Hz interval.

Overall, the SRFs are sharply focused within their respective frequency intervals and have balanced side lobes that are small relative to the peak, indicating high fidelity of the represented frequencies. The 0–10 Hz SRF has its peak at 0 Hz instead of the interval center because the interval largely corresponds to the PG encoding spectrum; however, achieving a peak closer to the center of this interval is infeasible because oscillations at such frequencies could not be completed within the 90 ms maximum encoding duration. The 50+ Hz SRF has its peak at 61 Hz and is broader than the other SRFs because its bandwidth is not constrained by the width of the frequency interval.

### 4.2 In vivo measurements

**FIGURE 5:**
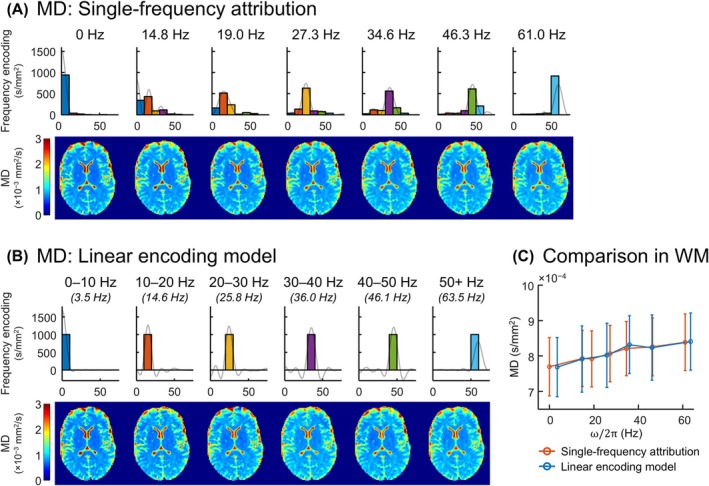
MD determined using different measurement representations. (A) MD corresponding to single‐frequency attribution. Above each MD map is the conventionally assigned frequency (0 Hz for PG, and the centroid of the encoding spectrum for others) in addition to the encoding coefficients for the linear encoding model. Encoding spectra (scaled by 10/π) are superimposed onto the encoding coefficient plots to illustrate the spectral lineshape for which the encoding coefficients were determined. (B) MD determined using the linear encoding model. Above each MD map is the corresponding frequency interval, both written and visualized in terms of its boxcar encoding representation and true spectral characterization (i.e., SRF scaled by the b‐value), as well as the centroid frequency of the interval's SRF. (C) Comparison of MD determined using both representations in WM. MD is shown in terms of the median and SD over WM for each point. Results of single‐frequency attribution are plotted to the assigned frequencies. Results of the linear encoding model are plotted to SRF centroid frequencies. MD, mean diffusivity; PG, pulsed gradient; SRF, spectral response function; WM, white matter.

Figure 5 illustrates the linear encoding model applied to MD measurements of one volunteer in terms of maps and plots of MD. The plot shows MD(ω) in WM within the slice depicted in the maps, and two trendlines are shown: one computed using single‐frequency attribution (0 Hz for PG, centroid of |*Q*(ω)|^2^ otherwise), and one computed using the linear encoding model. Equivalent plots depicting MD(ω) in global WM and global GM per volunteer are shown in Figure S4. Voxels with MD ≥ 2 × 10^−3^ mm^2^/s were excluded from consideration to mitigate CSF partial voluming. MD is seen to increase with increasing frequency using both representation paradigms. The frequency dependencies are very similar, but the agreement is not perfect because the two representations incorporate spectral information differently.

**FIGURE 6:**
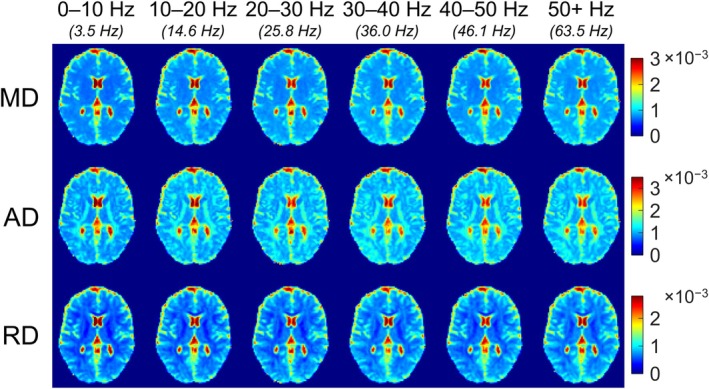
Representative maps of MD, AD, and RD over the fitted frequency intervals determined using the linear encoding model. The centroid frequency of each interval's SRF is indicated beneath the interval. AD, axial diffusivity; MD, mean diffusivity; RD, radial diffusivity; SRF, spectral response function.

**FIGURE 7:**
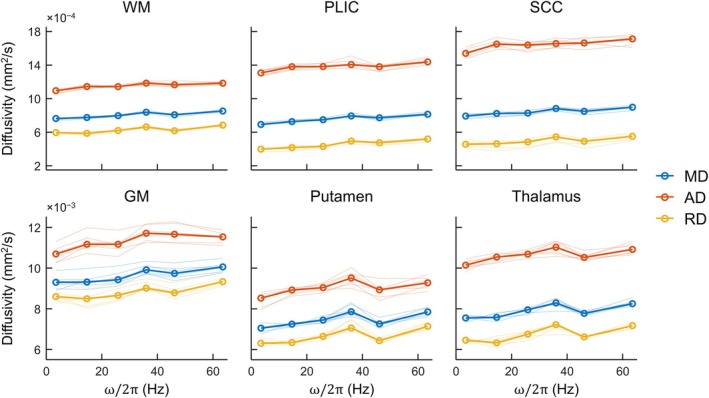
Frequency‐dependent curves of MD, AD, and RD in WM (top row) and GM (bottom row) regions. In each subplot, the bold curves show the median of each diffusivity across volunteers, computed using per‐volunteer medians of each diffusivity. The thinner, more transparent curves show the median of each diffusivity per volunteer. Vertical axes are consistent per row. Results were determined using the linear encoding model and are plotted to SRF centroid frequencies. AD, axial diffusivity; GM, gray matter; MD, mean diffusivity; PLIC, posterior limb of the internal capsule; RD, radial diffusivity; SCC, splenium of the corpus callosum; SRF, spectral response function; WM, white matter.

Further results using the linear encoding model for MD, AD, and RD are shown in Figure 6, where maps of each diffusivity in one volunteer are shown. Figure 7 shows plots of the three diffusivities for three WM regions (global WM, the posterior limb of the internal capsule, and the splenium of the corpus callosum) and three GM regions (global GM, the putamen, and the thalamus) in terms of the median per volunteer and among per‐volunteer medians. Diffusivities are higher in WM than in GM, and all diffusivities tend to increase with increasing frequency, as in Figure 5. Unexpectedly, however, there is persistent reduction in diffusivity from the 30–40 Hz to the 40–50 Hz frequency interval, which was observed to coincide with a recurring discontinuity—often also a dip—in the measurement diffusivity data (i.e., inputs to the linear encoding model), as can be seen in Figure S4. The systematic nature of the discontinuity suggests a nonbiological origin, and post hoc analysis ruled out b‐tensor cross‐terms resulting from spoiler gradients as a potential cause. Nonetheless, this behavior underscores that sharpening spectral response via the linear encoding model can amplify spectrally local anomalies. Despite the seemingly artifactual source of such anomalies in the data presented here, this local feature enhancement may be desirable in the study of fine spectral details.

### 4.3 Simulated measurements

**FIGURE 8:**
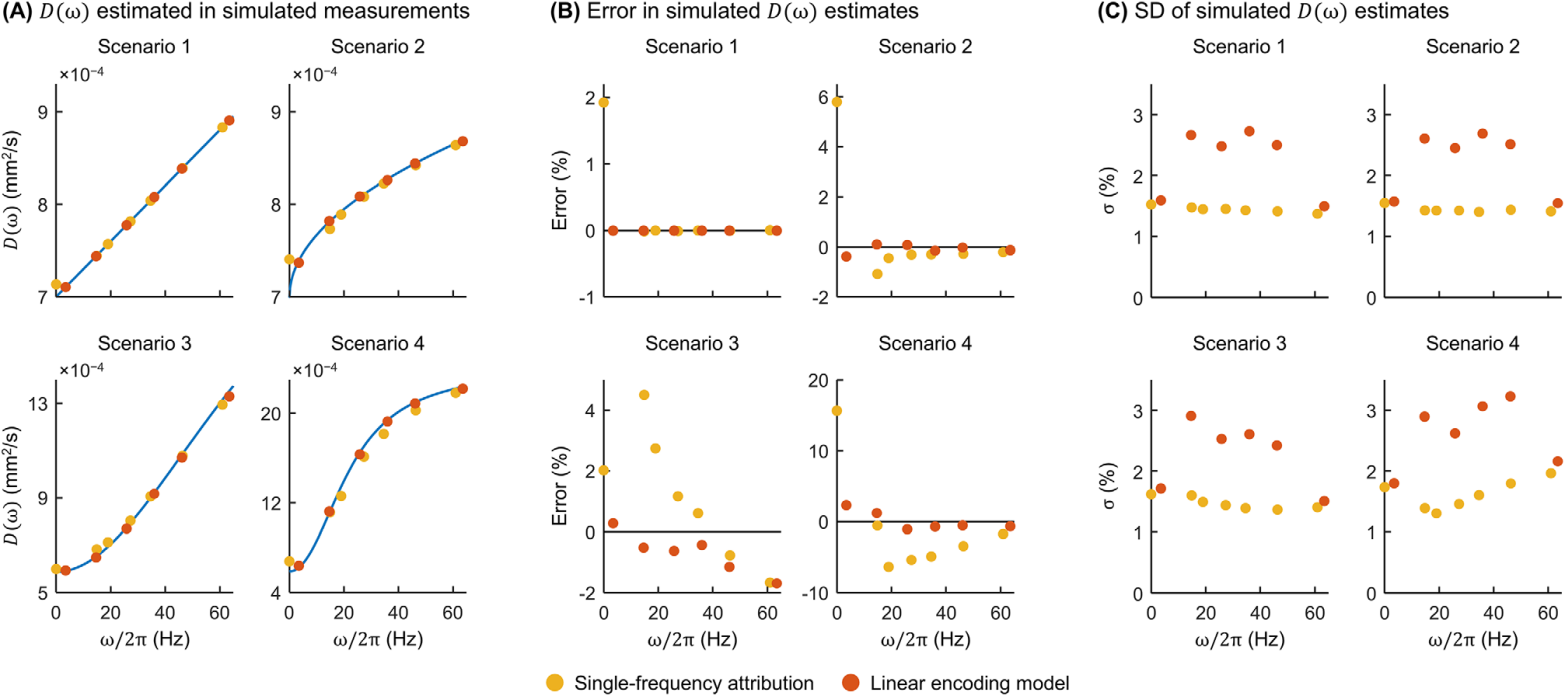
(A) Frequency‐dependent diffusivities from noiseless simulated measurements, as determined using single‐frequency attribution or the linear encoding model. Each plot represents a different theoretical microstructural scenario characterized by a known *D*(ω) (see Section 3.8). Simulated measurements of these spectra, shown as blue curves, yielded the frequency‐dependent diffusivity estimates, shown as dots. (B) Percentage errors of diffusivities estimated in (A) with respect to the ground truth. Ground truth *D*(ω) values were given by *D*(ω) at the centroid of the respective interval's SRF for the linear encoding model and by *D*(ω) at the assigned frequency for single‐frequency attribution. (C) SDs of diffusivities estimated in repeated noisy simulated measurements of the same *D*(ω) as in (A). SDs are given as a percentage of the ground truth. Noise levels in these simulated measurements were designed to mimic those encountered on a per‐voxel basis in vivo. Please note that vertical axis values differ between all subplots of (A) and (B). SRF; spectral response function.

In Figure 8, diffusivity estimation performance is shown for simulated measurements employing encodings matching the in vivo measurements. Figure 8A illustrates diffusion spectra corresponding to the four theoretical scenarios alongside diffusivities estimated using single‐frequency attribution and the linear encoding model in the noiseless simulated measurements. The errors in these estimates are given in Figure 8B. Figure 8C illustrates the SDs of diffusivity estimates in the presence of noise for both measurement representations. The errors for both representations are low on average but consistently lower using the linear encoding model: across all diffusion spectra, the average absolute error is 2.21% for single‐frequency attribution versus 0.50% for the linear encoding model, amounting to a 78% reduction. For both representations, error increases as *D*(ω) locally departs from linearity and is highest for scenarios 3 and 4, for which the diffusion spectra have the greatest changes in slope, but nonlinearities in *D*(ω) are better estimated using the linear encoding model. On the other hand, variability in estimated diffusivity is greater using the linear encoding model than using single‐frequency attribution, by 57% on average, and is relatively consistent for different *D*(ω).

In Table S1, errors and SDs of diffusion dispersion parameters estimated using both measurement representations are shown for the first two theoretical scenarios, using the same simulation data represented by Figure 8. Using the linear encoding model, all parameters were fitted with significantly improved accuracy and marginally reduced precision.

In Table 2, errors and SDs aggregated over all theoretical scenarios are tabulated for simulated measurements using spectral encodings optimized for different interval widths. Errors and SDs using single‐frequency attribution appear to be independent of interval width, considering that errors for the 25‐Hz and 16.7‐Hz interval simulations are likely artificially lower because PG sequences, which contribute anomalously high error (see Figure 8B), were not used. Errors using the linear encoding model are greatest for 25‐Hz intervals and lowest for 8.3‐Hz intervals but are relatively stable with respect to interval width overall, whereas SDs become increasingly elevated as interval width decreases. Notably, the actual interval widths—as defined by SRF FWHMs—only marginally increase as intervals nominally widen (Table S2); this behavior likely occurred because the same library containing |*Q*(ω)|^2^s with bandwidths down to ˜10 Hz was used for all nominal interval widths. Consequently, higher nominal resolution did not yield commensurate gains in per‐measurement specificity (i.e., accuracy) and limited the achievable orthogonality among encodings, thereby increasing measurement uncertainty. Nonetheless, the 10‐Hz nominal interval widths utilized in vivo most closely match the actual interval widths, thereby achieving maximal coverage without oversampling.

**TABLE 2:** Errors and SDs of diffusivity estimates in simulated frequency‐dependent diffusion measurements for different frequency interval widths. The *M* optimal encodings for each interval width and corresponding set of *N* frequency intervals were employed in the simulations (see Figure S3). As described in Section 3.8.1, errors derive from the noiseless simulated measurements, and SDs derive from the repeated noisy simulated measurements. Noise levels in the noisy simulated measurements were designed to mimic those encountered on a per‐voxel basis in vivo. For each interval width, the average absolute percentage error and the average SD as a percentage of the ground truth were taken over all encodings (for single‐frequency attribution) or intervals (for the linear encoding model) and all four theoretical *D*(ω). Ground truth *D*(ω) values were given by *D*(ω) at the centroid of the respective interval's SRF for the linear encoding model and by *D*(ω) at the assigned frequency for single‐frequency attribution. Tabulated values for the 10‐Hz intervals are based on the data depicted in Figure 8.

Width of primary frequency intervals	Measurement representation	Average absolute error (%)	Average SD (%)
25 Hz	Single‐frequency attribution	1.12	1.55
(*N* = 3, *M* = 3)	Linear encoding model	0.54	1.73
16.7 Hz	Single‐frequency attribution	0.98	1.53
(*N* = 4, *M* = 4)	Linear encoding model	0.50	1.80
12.5 Hz	Single‐frequency attribution	2.26	1.52
(*N* = 5, *M* = 5)	Linear encoding model	0.50	1.99
10 Hz	Single‐frequency attribution	2.21	1.49
(*N* = 6, *M* = 7)	Linear encoding model	0.50	2.35
8.3 Hz	Single‐frequency attribution	2.48	1.49
(*N* = 7, *M* = 8)	Linear encoding model	0.34	2.76

Abbreviations: SRF, spectral response function.

## 5 DISCUSSION

A linear encoding model for the measurement of frequency‐dependent diffusion was proposed in which spectral measurements are represented by encoding power in |*Q*(ω)|^2^ over a set of contiguous frequency intervals. The model was deployed in in vivo human brain imaging in accordance with an associated implementation framework concerning the selection of frequency intervals and |*Q*(ω)|^2^s used in measurements. The resulting diffusivity estimates were in close agreement with those of the conventional procedure of ascribing spectral measurements to single frequencies; this agreement was in line with simulated measurements of highly linear *D*(ω), for which single‐frequency attribution yielded accurate estimates of *D*(ω). Importantly, the simulated measurements demonstrated that the linear encoding model dependably achieved more accurate estimates of *D*(ω), especially for more nonlinear *D*(ω), at the expense of lower precision.

The key outcome of the proposed strategy is an enhanced set of response functions to which the resulting frequency‐dependent diffusivities can be mapped. Whereas conventional TDS limits response functions to the employed |*Q*(ω)|^2^s, the linear model constructs more selective response functions (i.e., SRFs) by exploiting encoding redundancies in said |*Q*(ω)|^2^s. Importantly, SRFs are constrained to have nulled spectral area outside of the intended frequency interval. As a result, SRFs have oscillations around zero and are not strictly positive, unlike |*Q*(ω)|^2^s. These wiggles come at the expense of noise amplification but provide a sharpening effect on SRFs^35^ that enables more accurate estimation of frequency‐dependent diffusivity. This improvement in accuracy can be expected to outweigh the reduction in precision, particularly when diffusion metrics are taken over ROIs, for which having more voxels diminishes uncertainty.

Although SRFs define the spectral responses corresponding to estimated frequency‐dependent diffusivities, boxcar functions represent diffusivity in the linear encoding model. This representation contrasts with conventional depiction of diffusion spectra, i.e., as curves. That said, diffusion spectra attained by the linear encoding model need not be imagined or depicted as boxcars. Because SRFs ultimately underlie the boxcars, plotting estimated diffusivities to SRF centroids, as was done here, is both faithful to reality and compatible with standard depiction of *D*(ω). In this way, frequency intervals are attributed to single frequencies, resulting in an apparent discrepancy with the aim of this work to overcome the limitations of single‐frequency attribution. However, because SRFs are more spectrally selective than |*Q*(ω)|^2^s, the assignment of a frequency interval to its SRF's centroid constitutes a higher‐fidelity representation. Furthermore, it is worth noting that the improved accuracy in estimating diffusivity was determined using this assignment.

Selection of encoding spectra in TDS experiments is typically ad hoc, where equidistant spacing of assigned frequencies is often preferred. The selection protocol in this work utilizes an optimization framework that factors noise and spectral contamination into the choice of encoding spectra, thereby introducing more tangible, quantitative considerations to the selection process. Previous works have also selected encoding spectra via optimization^10^, ^31^ but in a more limited context in which only two measurements were performed and *D*(ω) was assumed to follow a power law dependence.

An assumption on *D*(ω) is also implicitly made here as given by the noise amplification coefficients in the objective function. The constant signal noise level in all ln(*S*_0_/*S*_*m*_) assumed in Eq. 10 is only possible if all *S*_*m*_ have the same signal level. Because each *S*_*m*_ reflects different spectral selectivity, equivalence in signal levels requires that *D*(ω) is frequency‐independent. Because this assumption was not satisfied, the true noise amplification in simulations was slightly higher than predicted by Eq. 10.

The encoding spectra selection framework was developed heuristically to deliver key desirable characteristics for the ultimate frequency‐dependent diffusivity estimates. Further refinement of either the objective function or selection algorithm could improve encoding performance but was beyond the scope of this work. For instance, the quantities in the objective function could have been combined in a different fashion, or a different algorithm could have been used considering that the greedy strategy produces locally optimal solutions in a stepwise fashion but does not guarantee global optimality of the |*Q*(ω)|^2^s for all *M*. That said, both variants of the employed algorithm yielded comparable and satisfactory results, indicating the adequacy of the present framework and the final selection of encoding spectra.

The presented linear encoding model and sequence selection framework offer several benefits to the investigation of frequency‐dependent diffusivity. The measurement of diffusion spectra with improved accuracy and spectral resolution should aid efforts to validate theoretical diffusion models and, more generally, enable extraction of richer information from TDS. The use of narrower frequency intervals demands additional scan time and degrades precision but can be particularly advantageous in probing diffusion spectra with more rapid changes in slope with frequency. Furthermore, the 10–20 Hz interval sampled here spans a frequency range that is difficult to access by single‐frequency attribution due to constraints on the diffusion encoding duration. The capacity to measure this range with high selectivity can improve the evaluation of low‐frequency spectral features, which are present in many human tissues.^6^, ^36^ The effective mapping of the PG encoding spectrum to its centroid^37^ rather than to 0 Hz should also help in this regard, considering that 0 Hz is a consistent apparent misattribution as seen in Figure 8. Finally, the linear model offers a means to retrospectively disentangle spectral contributions from a series of frequency‐dependent diffusion measurements; such an implementation, however, would involve frequency‐interval selection as the second step and should be explored in future work.

## 6 CONCLUSION

The presented representation for spectral diffusion measurements based on interval‐wise encoding power enabled enhanced spectral response for the estimated diffusivities and, as a result, more accurate determination of *D*(ω). This encoding paradigm should prove increasingly beneficial in the characterization of diffusion spectra as the degree of nonlinearity in *D*(ω) increases.

## Supporting information


**Figure S1.** Spectral response functions (SRFs), noise amplification coefficients, and power in SRF side lobes for the optimal sets of encoding spectra for *M* = 6 through 9.
**Figure S2.** Ratio between actual and nominal b‐value per sequence and diffusion direction determined using phantom data and corrected for in in vivo data. Sequence numbers correspond to those given in Figure 4A and reflect which diffusion gradient waveform / encoding spectrum was used. Each plot line corresponds to a nominal diffusion gradient direction vector shown in the table on the right, for which positive values correspond to the left, anterior, and superior directions for the *x*, *y*, and *z* axes, respectively. AP, anterior–posterior; LR, left–right; SI, superior–inferior.
**Figure S3.** Encoding properties of diffusion gradients selected for different interval widths using the combinatorial optimization framework described in Section 3.3. In each subfigure heading, the stated interval width corresponds to the primary intervals up to 50 Hz, *N* is the number of frequency intervals, and *M* is the number of spectral diffusion measurements. The final selection of encoding spectra per *N* was performed in the same manner as described in Section 3.4, that is, by weighing the utility of reduced diffusivity estimation uncertainty and spectral contamination against increased measurement time with increasing *M*.
**Figure S4.** Comparison of frequency‐dependent mean diffusivity (MD) determined using single‐frequency attribution and the linear encoding model in global white matter (WM) and global gray matter (GM) for each volunteer. Each point shows the median of MD over the relevant segmented region. Results of single‐frequency attribution are plotted to the assigned frequencies, and results of the linear encoding model are plotted to SRF centroid frequencies. SRF, spectral response function.
**Table S1.** Accuracy and precision of diffusion dispersion metrics fitted to diffusivity estimates in simulated frequency‐dependent diffusionmeasurements. Errors and standard deviations (SDs) in this table are based on the simulated measurements represented by Figure 8, i.e., using the same spectral encodings as were used in the in vivo imaging experiments. Errors derive from the noiseless simulated measurements, and SDs derive from the repeated noisy simulated measurements. Errors and SDs are given as percentages of the ground truth values stated in Section 3.8.1. In least‐squares fitting of *D*_0_ and Λ based on *D*(ω) = *D*_0_ + Λω^θ^, ω values were given by SRF centroid frequencies for the linear encoding model and the conventional assigned frequencies (0 Hz for PG, centroid of |*Q*(ω)|^2^ otherwise) for single‐frequency attribution.
**Table S2.** Width of primary frequency intervals vs. the average FWHM of corresponding SRFs. The SRFs over which the averages were taken are shown in Figure S3 in different subfigures. The 50+ Hz interval and its SRF were excluded from consideration in all cases. FWHM, full width at half maximum; SRF, spectral response function.
