# Supplementary material for: Enhanced spectral response in frequency‐dependent diffusion measurements using a linear encoding model
Source: Magn Reson Med. 2025 Jul 28;94(6):2405–20. doi: 10.1002/mrm.70006 (PMC12501664; doi:10.1002/mrm.70006)
Supplement: Supplementary file 1 — Figure S1. Spectral response functions (SRFs), noise amplification coefficients, and power in SRF side lobes for the optimal sets of encoding spectra for M = 6 through 9. Figure S2. Ratio between actual and nominal b‐value per sequence and diffusion direction determined using phantom data and corrected for in in vivo data. Sequence numbers correspond to those given in Figure 4A and reflect which diffusion gradient waveform / encoding spectrum was used. Each plot line corresponds to a nominal diffusion gradient direction vector shown in the table on the right, for which positive values correspond to the left, anterior, and superior directions for the x, y, and z axes, respectively. AP, anterior–posterior; LR, left–right; SI, superior–inferior. Figure S3. Encoding properties of diffusion gradients selected for different interval widths using the combinatorial optimization framework described in Section 3.3. In each subfigure heading, the stated interval width corresponds to the primary intervals up to 50 Hz, N is the number of frequency intervals, and M is the number of spectral diffusion measurements. The final selection of encoding spectra per N was performed in the same manner as described in Section 3.4, that is, by weighing the utility of reduced diffusivity estimation uncertainty and spectral contamination against increased measurement time with increasing M. Figure S4. Comparison of frequency‐dependent mean diffusivity (MD) determined using single‐frequency attribution and the linear encoding model in global white matter (WM) and global gray matter (GM) for each volunteer. Each point shows the median of MD over the relevant segmented region. Results of single‐frequency attribution are plotted to the assigned frequencies, and results of the linear encoding model are plotted to SRF centroid frequencies. SRF, spectral response function. Table S1. Accuracy and precision of diffusion dispersion metrics fitted to diffusivity estimates in simulated frequency‐dependent [file MRM-94-2405-s001.pdf]

SUPPORTING INFORMATION FOR:

Enhanced spectral response in frequency-dependent diffusion measurements using a linear encoding model

Eric Seth Michael | Franciszek Hennel | Klaas Paul Pruessmann

Institute for Biomedical Engineering, ETH Zurich and University of Zurich, Zurich, Switzerland

Encoding spectrum library

Three forms of diffusion gradient waveforms were used in generating the library of encoding spectra: pulsed gradients, oscillating gradients, and double-bipolar gradients. Various realizations of each type of waveform were created by iteratively incrementing the key variable parameters, excluding gradient amplitude and ramp times (equivalently, slew rate), which were set to 200 mT/m and 0.5 ms, respectively. Every realization satisfying $b \geq 1000$ s/mm^2 and $T_{DW} \leq 90$ ms was kept and scaled down in amplitude as necessary to reach $b = 1000$ s/mm^2, then the corresponding encoding spectrum was added to the library.

For pulsed gradients, the variable parameters were the duration of each trapezoidal pulse, δ, and the duration between pulses from beginning to beginning, Δ. The iteration framework produced waveform realizations for all $\delta = 7 + 2d$ (ms) and $\Delta = 12 + 2D$ (ms) for which $\Delta \geq \delta + 5$ (ms), where d and D are non-negative integers. There were 342 such waveforms with $b \geq 1000$ s/mm^2 and $T_{DW} \leq 90$ ms.

For oscillating gradients, the variable parameters were the number of periods on each side of the refocusing RF pulse, n_{per}, the oscillation frequency, f, and the duration between pulses from end to beginning, T_{gap}. Standard 1+1-period oscillating gradient waveforms were used, where the waveform duration is given by $T_{DW} = 2n_{per}/f + T_{gap}$ and $n_{per} = 1$. The iteration framework produced waveform realizations for $f = 24 + 2F$ (Hz) and $T_{gap} = 5 + 2t$ (ms), where F and t are non-negative integers. Moreover, waveforms were produced for both matched and unmatched polarity of the two pulses. There were 318 such waveforms with $b \geq 1000$ s/mm^2 and $T_{DW} \leq 90$ ms.

Gap-filled oscillating gradient waveforms[1] in the flow-compensated configuration[2] were also used, where the waveform duration is given by $T_{DW} = (2n_{per} + 0.5)/f$. The iteration framework

produced waveform realizations for all $n_{\mathrm{per}} = 1 + p/2$, $f = 28 + 2F$ (Hz), and $T_{\mathrm{gap}} = 5 + 2t$ (ms) (where $T_{\mathrm{gap}} \leq 1/2f$), where p, F, and t are non-negative integers. There were 98 such waveforms with $b \geq 1000$ s/mm^2 and $T_{\mathrm{DW}} \leq 90$ ms.

For double-bipolar gradients, the variable parameters were the duration of each bipolar pulse, δ, and the duration between pulses from end to beginning, T_{gap}. The iteration framework produced waveform realizations for all $\delta = 14 + 2d$ (ms) and $T_{\mathrm{gap}} = 5 + 2t$ (ms), where d and t are non-negative integers. Moreover, waveforms were produced for both matched and unmatched polarity of the two pulses. There were 450 such waveforms with $b \geq 1000$ s/mm^2 and $T_{\mathrm{DW}} \leq 90$ ms.

Selection of encoding spectra for in vivo experiments

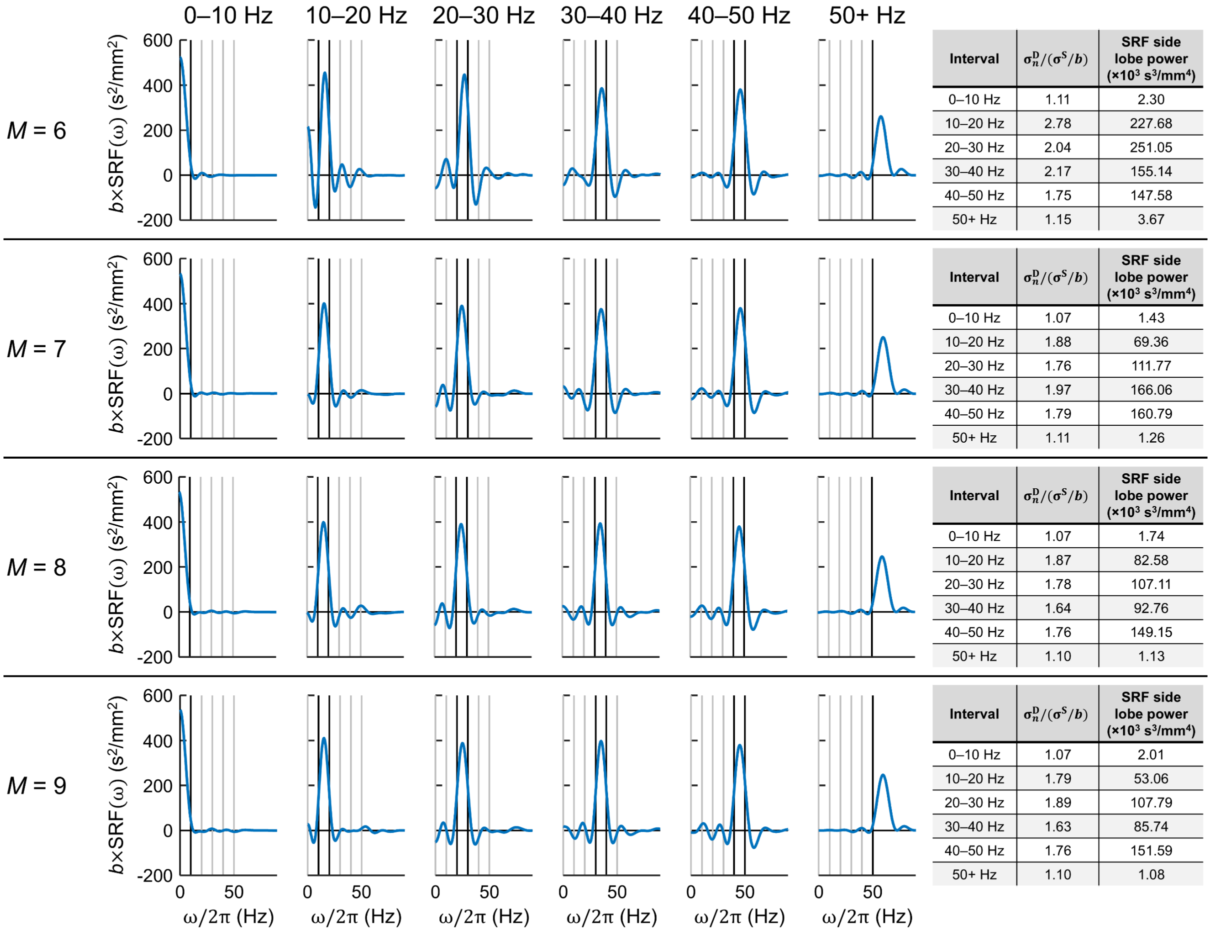

M = 6

Interval	$\sigma_n^{\mathrm{D}}/(\sigma^{\mathrm{S}}/b)$	SRF side lobe power (×10³ s³/mm⁴)
0–10 Hz	1.11	2.30
10–20 Hz	2.78	227.68
20–30 Hz	2.04	251.05
30–40 Hz	2.17	155.14
40–50 Hz	1.75	147.58
50+ Hz	1.15	3.67

M = 7

Interval	$\sigma_n^{\mathrm{D}}/(\sigma^{\mathrm{S}}/b)$	SRF side lobe power (×10³ s³/mm⁴)
0–10 Hz	1.07	1.43
10–20 Hz	1.88	69.36
20–30 Hz	1.76	111.77
30–40 Hz	1.97	166.06
40–50 Hz	1.79	160.79
50+ Hz	1.11	1.26

M = 8

Interval	$\sigma_n^{\mathrm{D}}/(\sigma^{\mathrm{S}}/b)$	SRF side lobe power (×10³ s³/mm⁴)
0–10 Hz	1.07	1.74
10–20 Hz	1.87	82.58
20–30 Hz	1.78	107.11
30–40 Hz	1.64	92.76
40–50 Hz	1.76	149.15
50+ Hz	1.10	1.13

M = 9

Interval	$\sigma_n^{\mathrm{D}}/(\sigma^{\mathrm{S}}/b)$	SRF side lobe power (×10³ s³/mm⁴)
0–10 Hz	1.07	2.01
10–20 Hz	1.79	53.06
20–30 Hz	1.89	107.79
30–40 Hz	1.63	85.74
40–50 Hz	1.76	151.59
50+ Hz	1.10	1.08

FIGURE S1. Spectral response functions (SRFs), noise amplification coefficients, and power in SRF side lobes for the optimal sets of encoding spectra for $M = 6$ through 9.

Sequence-wise b-value correction

The basis for sequence-wise b-value correction is that diffusivity should be frequency-independent (i.e., identical for all encoding spectra) and isotropic in the water phantom, assuming free diffusion. Results from the phantom measurements in conflict with these expectations were considered to be manifestations of system and gradient waveform imperfections and were corrected for.

For the phantom imaging data, voxel-wise diffusivities were first computed per diffusion direction using maps describing the spatial variation of the b-value to account for the effects of gradient nonlinearity during diffusion encoding. The b-value maps were dependent on the nominal diffusion direction but independent of the DTI sequence (i.e., gradient waveform / encoding spectrum) and were computed using vendor-provided gradient field distributions per Cartesian gradient axis.[3]

For each sequence, voxel-wise diffusivities were spatially averaged over all but the first two and final two slices of the phantom per diffusion direction. Subsequently, correction factors were computed per diffusion direction as

$$\Delta b_{i,m} = \frac{D_{i,m}}{D_{\mathrm{GT}}},$$

where $D_{i,m}$ denotes spatially averaged diffusivity along the ith diffusion direction for the mth DTI sequence (corresponding to diffusion gradient waveforms and encoding spectra in Figure 4A), and D_{GT} is the ground truth diffusivity. D_{GT} was taken as the powder average of the spatially averaged diffusivities for the PG sequence and was 2.23×10^{-3} mm^2/s.

For in vivo diffusion tensor fitting, spatially varying diffusion gradient direction vectors were scaled by the correction factor of the corresponding sequence and diffusion direction. As such, b-value correction per sequence and diffusion direction was performed. Correction factors are shown in Figure S2.

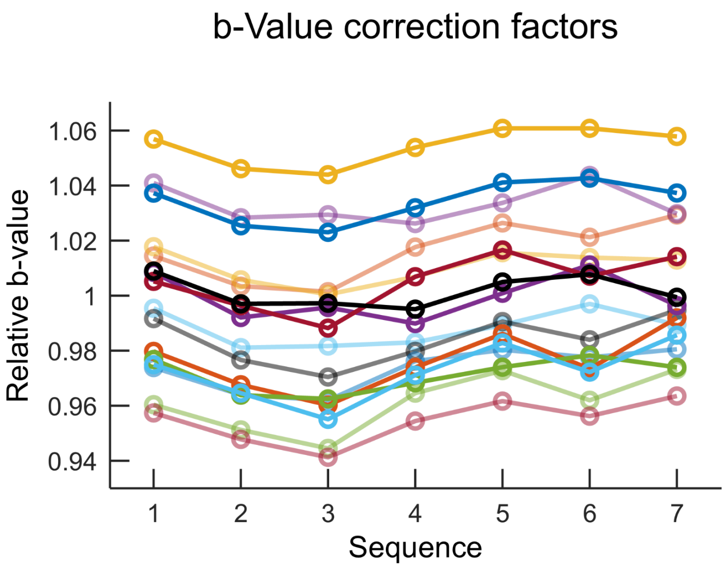

Color	*x* (LR)	*y* (AP)	*z* (SI)
○	0	0	1
○	0.814	0.371	0.447
○	0.604	−0.659	0.447
○	−0.410	0.448	0.795
○	−0.440	−0.778	0.447
○	−0.876	0.178	0.447
○	0.484	0.855	0.188
○	−0.552	−0.252	0.795
○	−0.664	0.724	0.188
○	0.595	−0.121	0.795
○	0.111	−0.976	0.188
○	−0.894	−0.407	0.188
○	−0.101	0.889	0.447
○	0.299	0.528	0.795
○	0.963	−0.196	0.188
○	0.069	−0.603	0.795

FIGURE S2. Ratio between actual and nominal b-value per sequence and diffusion direction determined using phantom data and corrected for in in vivo data. Sequence numbers correspond to those given in Figure 4A and reflect which diffusion gradient waveform / encoding spectrum was used. Each plot line corresponds to a nominal diffusion gradient direction vector shown in the table on the right, for which positive values correspond to the left, anterior, and superior directions for the *x*, *y*, and *z* axes, respectively. AP, anterior–posterior; LR, left–right; SI, superior–inferior.

Optimal encodings for different frequency interval widths

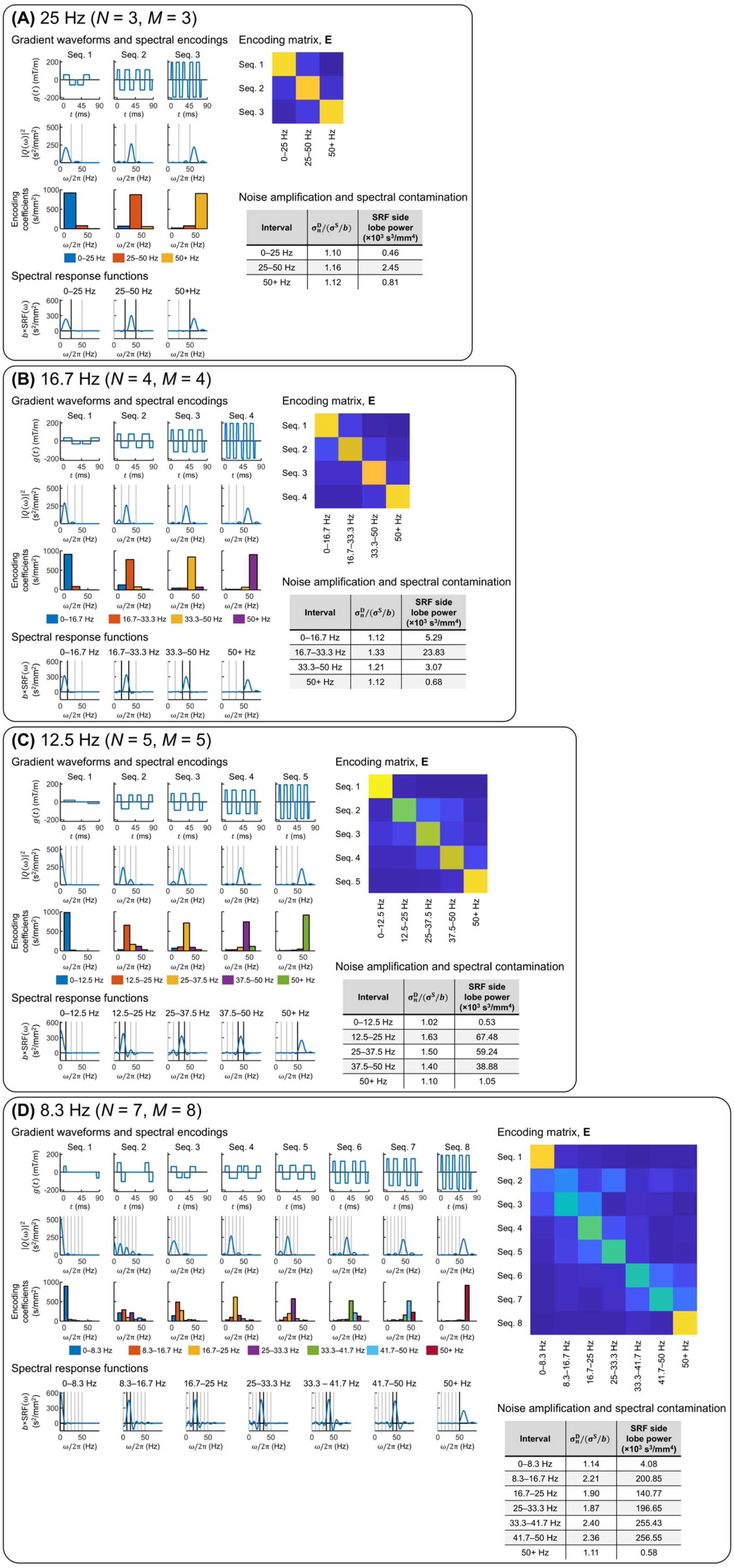

FIGURE S3. Encoding properties of diffusion gradients selected for different interval widths using the combinatorial optimization framework described in Section 3.3. In each subfigure

heading, the stated interval width corresponds to the primary intervals up to 50 Hz, N is the number of frequency intervals, and M is the number of spectral diffusion measurements. The final selection of encoding spectra per N was performed in the same manner as described in Section 3.4, that is, by weighing the utility of reduced diffusivity estimation uncertainty and spectral contamination against increased measurement time with increasing M.

MD(ω) per volunteer using both measurement representations

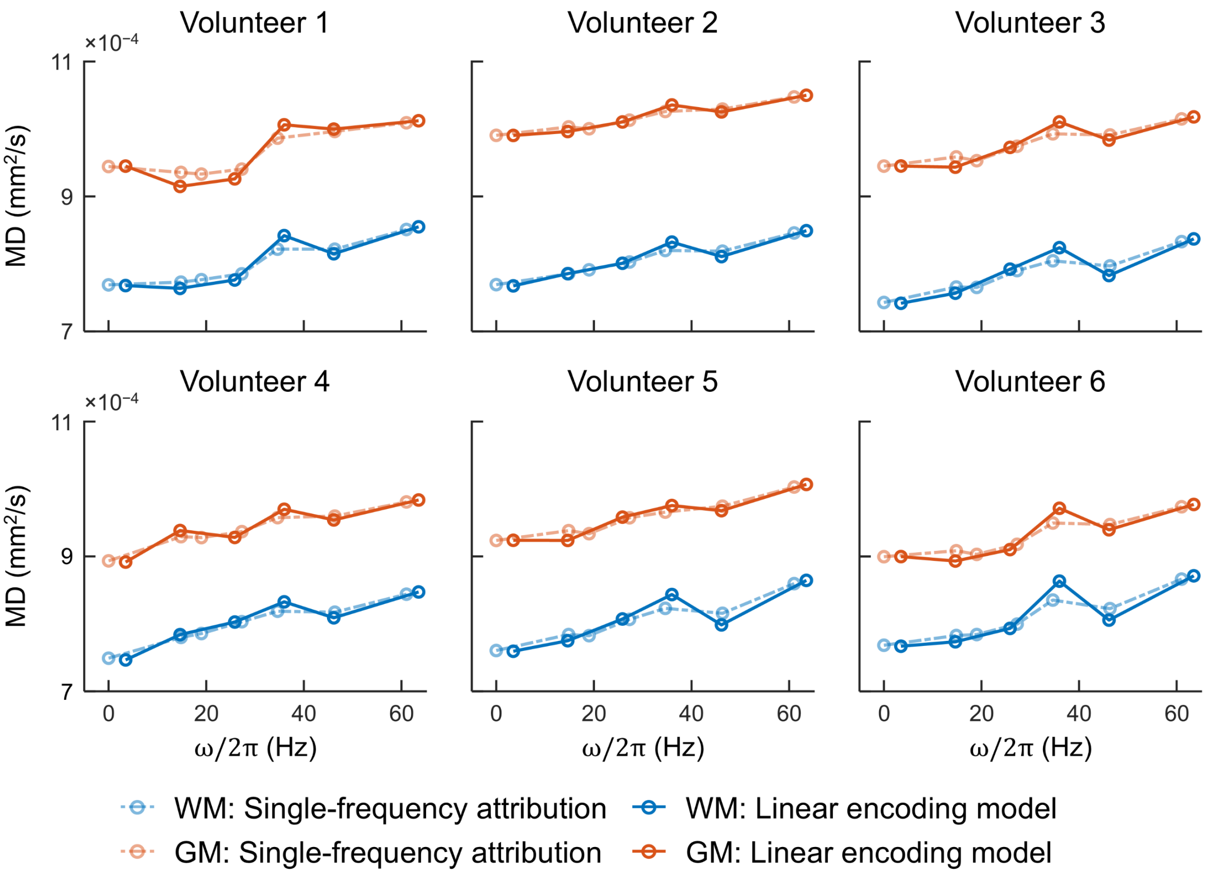

FIGURE S4. Comparison of frequency-dependent mean diffusivity (MD) determined using single-frequency attribution and the linear encoding model in global white matter (WM) and global gray matter (GM) for each volunteer. Each point shows the median of MD over the relevant segmented region. Results of single-frequency attribution are plotted to the assigned frequencies, and results of the linear encoding model are plotted to SRF centroid frequencies. SRF, spectral response function.

Diffusion dispersion parameter estimation using both measurement representations

TABLE S1. Accuracy and precision of diffusion dispersion metrics fitted to diffusivity estimates in simulated frequency-dependent diffusion measurements. Errors and standard deviations (SDs) in this table are based on the simulated measurements represented by Figure 8, i.e., using the same spectral encodings as were used in the in vivo imaging experiments. Errors derive from the noiseless simulated measurements, and SDs derive from the repeated noisy simulated measurements. Errors and SDs are given as percentages of the ground truth values stated in Section 3.8.1. In least-squares fitting of D_0 and Λ based on $D(\omega) = D_0 + \Lambda\omega^{\theta}$, ω values were given by SRF centroid frequencies for the linear encoding model and the conventional assigned frequencies (0 Hz for PG, centroid of $|Q(\omega)|^2$ otherwise) for single-frequency attribution.

	**Measurement representation**	***D*₀ (Scenario 1)**	**Λ (Scenario 1)**	***D*₀ (Scenario 2)**	**Λ (Scenario 2)**
**Error (%)**	Single-frequency attribution	0.92	−5.18	3.99	−24.42
	Linear encoding model	0	0	−0.22	0.84
**SD (%)**	Single-frequency attribution	1.16	7.38	1.39	8.19
	Linear encoding model	1.24	7.95	1.81	11.18

Nominal vs. actual frequency interval widths

TABLE S2. Width of primary frequency intervals vs. the average FWHM of corresponding SRFs. The SRFs over which the averages were taken are shown in Figure S3 in different subfigures. The 50+ Hz interval and its SRF were excluded from consideration in all cases. FWHM, full width at half maximum; SRF, spectral response function.

Width of frequency intervals (Hz)	Average SRF FWHM (Hz)
25	11.81
16.7	10.17
12.5	10.82
10	9.77
8.3	9.15

References

1. Hennel F, Michael ES, Pruessmann KP. Improved gradient waveforms for oscillating gradient spin-echo (OGSE) diffusion tensor imaging. *NMR Biomed*. 2021;34:e4434.
2. Michael ES, Hennel F, Pruessmann KP. Evaluating diffusion dispersion across an extended range of b-values and frequencies: Exploiting gap-filled OGSE shapes, strong gradients, and spiral readouts. *Magn Reson Med*. 2022;87:2710-2723.
3. Bammer R, Markl M, Barnett A, et al. Analysis and generalized correction of the effect of spatial gradient field distortions in diffusion-weighted imaging. *Magn Reson Med*. 2003;50:560-569.
